# Age-associated changes in human CD4^+^ T cells point to mitochondrial dysfunction consequent to impaired autophagy

**DOI:** 10.18632/aging.102438

**Published:** 2019-11-09

**Authors:** Arsun Bektas, Shepherd H. Schurman, Marta Gonzalez-Freire, Christopher A. Dunn, Amit K. Singh, Fernando Macian, Ana Maria Cuervo, Ranjan Sen, Luigi Ferrucci

**Affiliations:** 1 Translational Gerontology Branch, National Institute on Aging, National Institutes of Health, Baltimore, MD 21224, USA; 2 Clinical Research Branch, National Institute of Environmental Health Sciences, National Institutes of Health, Research Triangle Park, NC 27709, USA; 3 Flow Cytometry Unit, National Institute on Aging, National Institutes of Health, Baltimore, MD 21224, USA; 4 Laboratory of Molecular Biology and Immunology, National Institute on Aging, National Institutes of Health, Baltimore, MD 21224, USA; 5 Department of Pathology, Albert Einstein College of Medicine, Bronx, NY 10461, USA; 6 Institute for Aging Studies, Albert Einstein College of Medicine, Bronx, NY 10461, USA; 7 Department of Developmental and Molecular Biology, Albert Einstein College of Medicine, Bronx, NY 10461, USA

**Keywords:** proteomics, mitochondria, autophagy, aging, CD4^+^ T cells

## Abstract

To gain understanding on the mechanisms that drive immunosenescence in humans, we examined CD4^+^ T cells obtained from younger (20-39 years-old) and older (70+ years-old) healthy participants of the Baltimore Longitudinal Study on Aging (BLSA). We found that mitochondrial proteins involved in the electron transport chain were overrepresented in cells from older participants, with prevalent dysregulation of oxidative phosphorylation and energy metabolism molecular pathways. Surprisingly, gene transcripts coding for mitochondrial proteins pertaining to oxidative phosphorylation and electron transport chain pathways were underrepresented in older individuals. Paralleling the observed decrease in gene expression, mitochondrial respiration was impaired in CD4^+^ T cells from older subjects. Though mitochondrial number in both naïve and memory cells visualized with electron microcopy was similar in older versus younger participants, there were a significantly higher number of autophagosomes, many of them containing undegraded mitochondria, in older individuals. The presence of mitochondria inside the accumulated autophagic compartments in CD4^+^ T cells from older individuals was confirmed by immunofluorescence. These findings suggest that older age is associated with persistence of dysfunctional mitochondria in CD4^+^ T lymphocytes caused by defective mitochondrial turnover by autophagy, which may trigger chronic inflammation and contribute to the impairment of immune defense in older persons.

## INTRODUCTION

Human aging is characterized by increased susceptibility to diseases and impaired capacity to handle stress and challenges from the environment, leading to excess morbidity and mortality [1, 2]. A critical factor in this process is a progressive decline of immunity, which results in reduced protection against external microorganismal threats, diminished surveillance and reduced repair capacity of damaged cells and tissues that can lead to degenerative diseases. Indeed, rates of hospitalizations with a discharge diagnosis of infection rise dramatically with age, from 74 to 86 per 10,000 in individuals age 20–39 years-old to between 432 and 891 per 10,000 persons for those who are 70 years-old and older [3].

Changes in cellular morphology and function that occur in the immune system with aging are globally referred to as “immunosenescence” and have been widely studied and discussed in the literature with evidence that they involve both innate and adaptive immunity [4, 5]. However, the core mechanisms leading to these changes are unknown [6]. Two contrasting phenotypes characterize immunosenescence, a status of chronically activated mild inflammation, which has been termed “inflammaging”, and a progressive reduction of the ability to mount an adequate immune response to vaccination and to infection [6]. Inflammaging is characterized by a robust increase with aging of blood levels of proinflammatory markers, and is a strong risk factor for the occurrence, progression, and complication of many chronic diseases, including cardiovascular and neurodegenerative diseases [7, 8]. Several aspects of the immune response are affected by aging, including proper initiation and, especially, maintenance and cessation [6].

Recently, it has been proposed that most, if not all the aspects of the aging process, may be attributable to a dysfunction of a limited number of molecular and cellular mechanisms with aging, that have been called the “Hallmarks” or “Pillars” of aging [9, 10].

The “Pillars” of aging paradigm has not been systematically applied to the study of immunosenescence. One aspect that is increasingly being recognized as important to explain the effect of aging on immune cells, particularly CD8^+^ T cells, is the role of one of the components responsible for cellular proteostasis, macroautophagy (hereafter referred to as autophagy) and one of its modalities, mitophagy [11]. Studies in CD8^+^ T cells of older individuals have shown lower basal autophagy levels [12], while autolysosomes accumulate in human CD8^+^ T cells undergoing replicative senescence “in vitro” [13]. In CD4^+^ T cells, it has also been reported that while autophagy activity is lower in cells from older individuals, it is preserved in cells from the progeny of centenarians, which correlates with improved function [14]. Experiments of T-cell-specific depletion of *Vps34* [15] or *Atg7* [16], essential genes for canonical autophagy, lead to accumulation of damaged mitochondria and ROS, showing that T cells depend on autophagy and mitophagy for homeostasis and function [17]. Several lines of evidence suggest that defective autophagy may impair the recycling of dysfunctional mitochondria and profoundly affects the functionality of T lymphocytes [18, 19]. Naïve T cells that encounter a new antigen undergo extensive proliferation and differentiate into effector T cells. The increased energetic demand is supported by a metabolic shift to aerobic glycolysis, also called the “Warburg effect” [20]. Mitochondria also contribute to this process by using TCA cycle metabolites for the building of macromolecules, including proteins and lipids as building bricks for new cells, and by producing ROS signaling required for full T cell activation [21–23]. At infection resolution, most T cells undergo apoptosis while a few remodel into memory T cells. In spite of low energy consumption at rest, memory T cells display a characteristic increase in mitochondrial mass, which creates a large reserve respiratory capacity that allows for rapid and sustained proliferation upon secondary exposure to antigen reactivation [24]. Under the hypothesis that age impairs mitochondrial function in lymphocytes because of defective autophagy, the resulting energy deficit may impair both the activation of naïve T lymphocytes and reactivation of memory lymphocytes upon a second encounter with the same antigen, which are both critical response mechanisms of adaptive immunity [25]. This is consistent with the blunted response to influenza after vaccination in older persons, and the finding that treatment with an analog of rapamycin (RAD001), which stimulates autophagy, effectively restores, at least in part, such a response [26, 27].

To gain further understanding on the causes of immunosenescence, we compared CD4^+^ T cells from younger donors (ages 20-39 years-old) and older (ages 70 years and older) participants of the Baltimore Longitudinal Study of Aging (BLSA). In quantitative discovery proteomics of cytoplasmic extracts, we found that proteins related to oxidative phosphorylation and integration of energy metabolism were overrepresented in CD4^+^ T cells from older compared to younger individuals. At the same time, gene expression analysis showed that pathways related to oxidative phosphorylation and the electron transport chain were downregulated and mitochondrial respiration was impaired in cells from older compared to younger participants. Interestingly, transmission electron microscopy showed no difference in the number of mitochondria in both naïve and memory CD4^+^ T cells from younger and older participants, but substantial differences of mitochondrial morphology. In particular, many of the mitochondria from naïve and memory CD4^+^ T cells from older participants were morphologically distorted and enclosed into autophagosome vesicles. Autophagic flux assays confirmed reduced autophagy efficiency, suggesting that in older persons defective autophagy may impair the recycling of irreversibly damaged mitochondria leading to buildup of OXPHOS proteins. We propose that interventions that normalize autophagy may slow down immunosenescence and prevent its deleterious consequences.

## RESULTS

### Protein expression in human CD4^+^ T Cells

**Figure 1:**
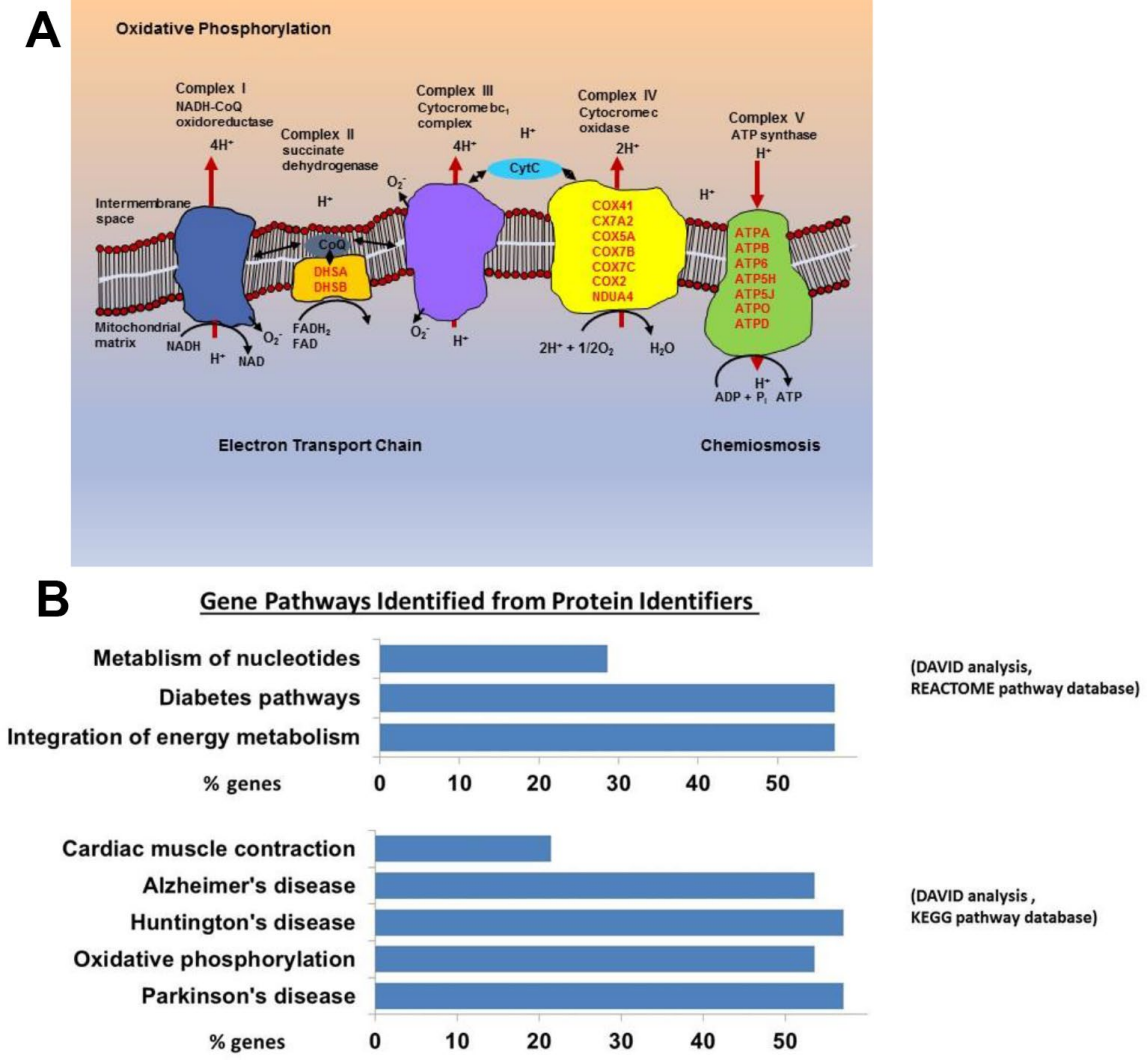
(**A**) Mitochondrial electron transport chain proteins that we found to be more highly expressed in the old are indicated in red by their location (Complex II, IV, and V) in the cartoon schema. (**B**) Gene pathways identified for proteins upregulated in the old using DAVID to convert protein identifiers to their genes (DAVID Bioinformatics Resources 6.7 (https://david.ncifcrf.gov). These genes fall into pathways related to oxidative phosphorylation, neurodegenerative disease, and integration of energy metabolism (Oxidative phosphorylation [16 from KEGG pathway database], Neurodegenerative disease [16 from KEGG], Cardiac muscle contraction [6 from KEGG], Integration of energy metabolism [16 from REACTOME pathway database], Diabetes [16 from REACTOME], Metabolism of nucleotides [8 from REACTOME]).

We examined the protein profiles from cytoplasmic extracts of human CD4^+^ T cells obtained from young (21-34 years-old) and older (68–83 years-old) male participants of the BLSA (Supplementary Table 1). Total CD4^+^ T cells were isolated from apheresis-derived peripheral blood mononuclear cells (PBMCs). Total CD4^+^ T lymphocytes, without distinction between naïve and memory subclass, were used for protein expression analysis because of the high number of cells needed to obtain enough cytoplastic extract to perform the proteomic study. The proportion of naïve CD4^+^ T cells (based on CD45RA^+^ expression) in the younger or older groups were comparable (Supplementary Figure 1), reflecting earlier published observations [28, 29]. Cytoplasmic extracts from CD4^+^ T cells were labeled with isobaric tags for relative and absolute quantification (iTRAQ) of proteins by liquid chromatography-mass spectrometry (LC-MS/MS). We compared 18 cytoplasmic extracts from older participants against four extracts from younger participants using four different iTRAQ 8plex kits. Equal amounts of cytoplasmic extract were used. One control sample from young participants was included in all four iTRAQ sets and used for protein quantitative normalization. To enhance comparability, technical controls (duplicate samples) were included within the same iTRAQ and also between iTRAQs (Supplementary Table 1). About 3,510 proteins were identified from each iTRAQ experiment. Data were analyzed within each iTRAQ 8plex and also in combination. Twenty-nine proteins (Table 1), ranked by significance (only proteins with *p* < 0.05 shown), were found to be up-regulated (ratio >1.3) in CD4^+^ T cells from older donors compared to younger donors after meeting additional analytical criteria (≥ 2 unique peptides, high confidence of the peptide sequences, and ion score of more than 30; see Materials and Methods). Interestingly, 23 of these were mitochondrial proteins (16 belonged to the electron transport chain; Figure 1A and Supplementary Table 2).

**Table 1:** Higher protein expression in older donors compared to younger donors as assessed by iTRAQ and mass spectrometry (LC-MS-MS) determined by high ratios (averages [old] / averages [young]) of protein expression in CD4^+^ T cells.

**Gene symbol**	**Gene description**	**Uniprot ID**	**Peptides description**	***p*-value**	**Average (y)**	**Average (o)**	**Ratio (o/y)**
GPDM	GPDM_HUMAN	P43304	(P43304) Glycerol-3-phosphate dehydrogenase, mitochondrial	0.0004	0.9734	1.5531	1.5956
HNRPL	HNRPL_HUMAN	P14866	(P14866) Heterogeneous nuclear ribonucleoprotein L	0.0006	1.1832	1.7053	1.4412
NB5R3	NB5R3_HUMAN	P00387	(P00387) NADH-cytochrome b5 reductase 3	0.0011	1.1119	1.5432	1.3879
COX41	COX41_HUMAN	P13073	(P13073) Cytochrome c oxidase subunit 4 isoform 1, mitochondrial	0.0012	1.0433	1.6018	1.5353
DHSB	DHSB_HUMAN	P21912	(P21912) Succinate dehydrogenase [ubiquinone] iron-sulfur subunit, mitochondrial	0.0018	0.9829	1.4623	1.4878
ATPB	ATPB_HUMAN	P06576	(P06576) ATP synthase subunit beta, mitochondrial	0.0019	1.0133	1.3217	1.3043
ATPA	ATPA_HUMAN	P25705	(P25705) ATP synthase subunit alpha, mitochondrial	0.0023	0.9533	1.2703	1.3325
NDUA4	NDUA4_HUMAN	O00483	(O00483) NADH dehydrogenase [ubiquinone] 1 alpha subcomplex subunit 4	0.0025	1.0289	1.5362	1.4931
SUN2	SUN2_HUMAN	Q9UH99	(Q9UH99) SUN domain-containing protein 2	0.0031	1.1153	1.5392	1.3801
COX5A	COX5A_HUMAN	P20674	(P20674) Cytochrome c oxidase subunit 5A, mitochondrial	0.0053	1.0940	1.7087	1.5619
MPCP	MPCP_HUMAN	Q00325	(Q00325) Phosphate carrier protein, mitochondrial	0.0057	1.1626	2.0633	1.7747
CYB5B	CYB5B_HUMAN	O43169	(O43169) Cytochrome b5 type B, mitochondrial	0.0082	1.1481	1.4972	1.3040
COX7B	COX7B_HUMAN	P24311	(P24311) Cytochrome c oxidase subunit 7B	0.0084	1.0530	2.0184	1.9168
PGRC2	PGRC2_HUMAN	O15173	(O15173) Membrane-associated progesterone receptor component 2	0.0104	1.0354	1.7378	1.6784
DHSA	DHSA_HUMAN	P31040	(P31040) Succinate dehydrogenase [ubiquinone] flavoprotein subunit, mitochondrial	0.0123	0.9902	1.5637	1.5792
BCL2	BCL2_HUMAN	P10415	(P10415) Apoptosis regulator Bcl-2	0.0125	1.0076	1.4705	1.4594
ATPD	ATPD_HUMAN	P30049	(P30049) ATP synthase subunit delta, mitochondrial	0.0148	0.9403	1.4305	1.5213
ATP6	ATP6_HUMAN	P00846	(P00846) ATP synthase subunit a, mitochondrial	0.0153	0.9667	1.6244	1.6804
COX7C	COX7C_HUMAN	P15954	(P15954) Cytochrome c oxidase subunit 7C, mitochondrial	0.0165	0.9625	2.0954	2.1770
CX7A2	CX7A2_HUMAN	P14406	(P14406) Cytochrome c oxidase subunit 7A2, mitochondrial	0.0177	1.0284	1.6805	1.6341
STML2	STML2_HUMAN	Q9UJZ1	(Q9UJZ1) Stomatin-like protein 2, mitochondrial	0.0260	1.0155	1.4892	1.4665
ITAM	ITAM_HUMAN	P11215	(P11215) Integrin alpha-M	0.0303	0.9807	1.3973	1.4248
COX2	COX2_HUMAN	P00403	(P00403) Cytochrome c oxidase subunit 2	0.0304	1.0580	1.4211	1.3432
ATP5H	ATP5H_HUMAN	O75947	(O75947) ATP synthase subunit d, mitochondrial	0.0345	1.0268	1.3764	1.3405
LMNB1	LMNB1_HUMAN	P20700	(P20700) Lamin-B1	0.0391	1.3814	1.9963	1.4451
ATP5J	ATP5J_HUMAN	P18859	(P18859) ATP synthase-coupling factor 6, mitochondrial	0.0431	1.0240	1.9787	1.9323
ATPO	ATPO_HUMAN	P48047	(P48047) ATP synthase subunit O, mitochondrial	0.0453	1.0983	1.6420	1.4950
ADT1	ADT1_HUMAN	P12235	(P12235) ADP/ATP translocase 1	0.0543	1.2815	1.7950	1.4007
DPM1	DPM1_HUMAN	O60762	(O60762) Dolichol-phosphate mannosyltransferase	0.0222	1.1323	1.6071	1.4193

Averages of ratios were calculated between old and young (averages [old] / averages [young]) after normalization using the common control sample used across iTRAQ 8plex experiments.

Using the Database for Annotation, Visualization and Integrated Discovery (DAVID), we found that proteins increased in CD4^+^ T cells from older participants mapped to several molecular pathways, namely oxidative phosphorylation (16 from KEGG pathway database), cardiac muscle contraction (6 from KEGG), integration of energy metabolism (16 from REACTOME pathway database), and metabolism of nucleotides (8 from REACTOME) (Figure 1B). These data demonstrate that mitochondrial proteins and particularly proteins that pertain to electron transport chain complexes accumulate with age in CD4^+^ T lymphocytes.

### Gene expression in human CD4^+^ T Cells

**Figure 2:**
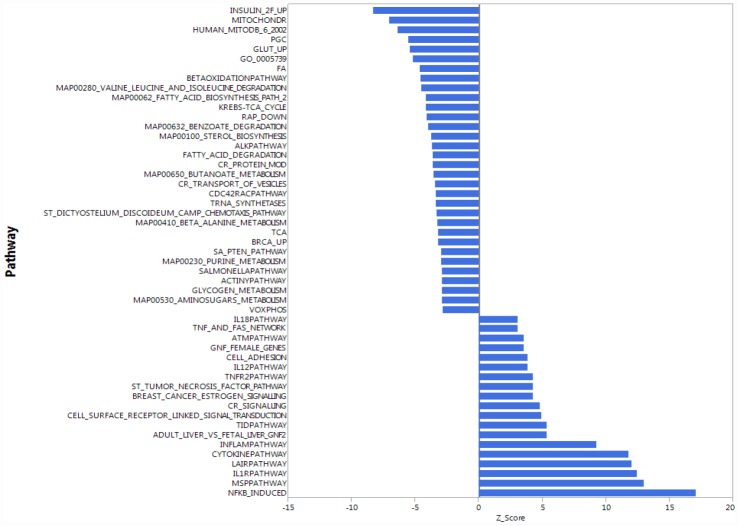
**Gene expression analysis showing top 51 up- and down-regulated biological pathways in CD4^+^ T cells between older (70 to 80 years-old) and younger (20 to 30 years-old) men.** Mitochondria-related and oxidative phosphorylation-related pathways were the most down-regulated pathways in older compared to younger donors. Differences of Z-scores between younger and older participants are shown on the X-axis. Each row denotes a different pathway (*p* ≤ 0.05 and FDR ≤ 0.3). N = 5 young, 19 old donors.

To understand whether the higher accumulation of mitochondrial proteins in the cytoplasm of CD4^+^ T lymphocytes from older compared to younger participants was due to increased transcription/translation or rather to defective elimination, we studied gene expression in human CD4^+^ T cells by using the Parametric Analysis of Gene Enrichment (PAGE) analysis [29, 30] of normalized hybridization data from cRNA. Cells from 33 donors were analyzed divided into a young group (5 men and 3 women, ages 25–38 years-old) and an older group (19 men and 6 women, ages 70–82 years-old), (Supplementary Table 3). Expression pathways were transformed into Z-scores, and significant differences (*p*-value ≤ 0.05) between young (Y) and old (O) donors were identified (Figure 2, Supplementary Figure 2A, 2B).

To be consistent with proteomic data, we first compared gene expression only between young and old men. Contrary to our proteomic analysis, PAGE identified “mitochondria-related” and “oxidative phosphorylation-related” pathways were amongst the most down-regulated biological pathways in older compared to younger individuals (Figure 2). That is, for many proteins found to be overrepresented in older persons CD4^+^ T lymphocytes, gene transcripts were instead underrepresented in CD4^+^ T lymphocytes from older persons. The same trends were noted when the analysis was restricted to women or included both sexes (Supplementary Figure 2A, 2B). Thus, proteomic and gene expression results tended to be inversely correlated.

### Differences of mitochondrial respiration between young and old CD4^+^ T Cells

To understand whether overrepresentation of mitochondrial proteins in CD4^+^ T lymphocytes from older compared to younger individuals comes from functional or dysfunctional mitochondria, we measured mitochondrial respiration in CD4^+^ T cells of 7 young (ages 22–35 years-old) and 7 old (ages 80–93 years-old) male participants (Supplementary Table 4A) using high-resolution respirometry OROBOROS Oxygraph-2k (O2k). We challenged CD4^+^ T lymphocytes from younger and older participants with several mitochondrial inhibitors in sequence to estimate mitochondrial reserve capacity, nonmitochondrial oxygen capacity, ATP-linked respiration and proton leak and we used these values to derive the Bioenergetic Health Index (BHI), a summary index of mitochondrial function in cells [31]. The BHI is derived from calculating a ratio of positive aspects of mitochondrial bioenergetic function (i.e. reserve capacity and ATP-linked respiration) to potentially deleterious aspects of mitochondrial bioenergetic function (i.e. non-mitochondrial oxygen consumption and proton leak) [31].

**Figure 3:**
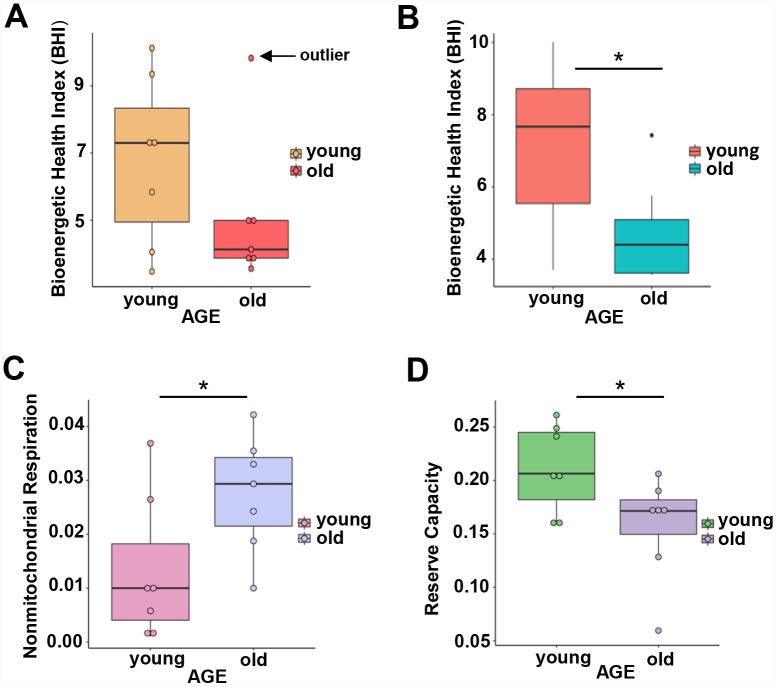
(**A**) Calculated bioenergetic health index (BHI) from young and old CD4^+^ T cells. The BHI is derived from calculating a ratio of positive aspects of mitochondrial bioenergetic function (i.e. reserve capacity and ATP-linked respiration) to potentially deleterious aspects of mitochondrial bioenergetic function (i.e. non-mitochondrial oxygen consumption and proton leak). Cellular mitochondrial function was determined using high-resolution respirometry with oligomycin, FCCP, rotenone, and antimycin A. For BHI, one outlier of 14 participants was noted and a comparison of the old and young BHI with this outlier was not significantly different (*p*=0.19). (**B**) In further analysis, FACS showed the outlier subject had a higher percentage of total memory CD4^+^ T cells (59% compared to a range 26-48%) than other participants. Adjusting for the average percentage of memory CD4^+^ T cells, the calculated the BHI was significantly higher for younger compared to older participants (*p = 0.036). (**C**) Nonmitochondrial respiration was found to be significantly higher in CD4^+^ T cells from older compared to younger individuals (*p = 0.049). (**D**) Reserve capacity was significantly higher in cells from young compared to older participants (*p = 0.045). (**A**–**D**) *P*-values were calculated by Welch’s t-test, a variation of the Student’s t-test that does not make the assumption of equal variance in the two compared samples [55]. Error bars reflect the standard error of the mean (±SEM). N = 7 young, 7 old donors.

BHI was higher in cells from younger compared to older participants (Supplementary Figure 3, representative mitochondrial respiration measurements), except for one outlier whose flow cytometric profile was different from the other 13 samples and the percentage of memory cells (central and effector) much higher than that percentage in the other 6 older donors (59% compared to a range 26–48%) (Figure 3A and Supplementary Table 4B). After accounting for differences due to different percent of memory cells between groups, the BHI differences between cells from younger and older subjects was statistically significant (*p* = 0.036) (Figure 3B). Of note, the majority (85%) of CD4^+^ T cells from younger participants had higher BHI compared to cells from older donors. Conversely, nonmitochondrial respiration, typically attributed to the action of cyclooxygenases, lipoxygenases and NADPH oxidases that are considered negative indicators of bioenergetic health, was significantly higher (*p* = 0.049) in CD4^+^ T lymphocytes from older compared to younger participants [31] (Figure 3C). We also found that the reserve mitochondrial capacity was significantly higher (*p* = 0.045) in cells from younger compared to older participants (Figure 3D). Cumulatively, these observations demonstrate that despite increased abundance of mitochondrial proteins, mitochondrial function of CD4^+^ lymphocytes is lower in older compared to younger individuals.

### Morphology of mitochondria and autophagic vacuoles in CD4^+^ T cells from young and old participants

**Figure 4:**
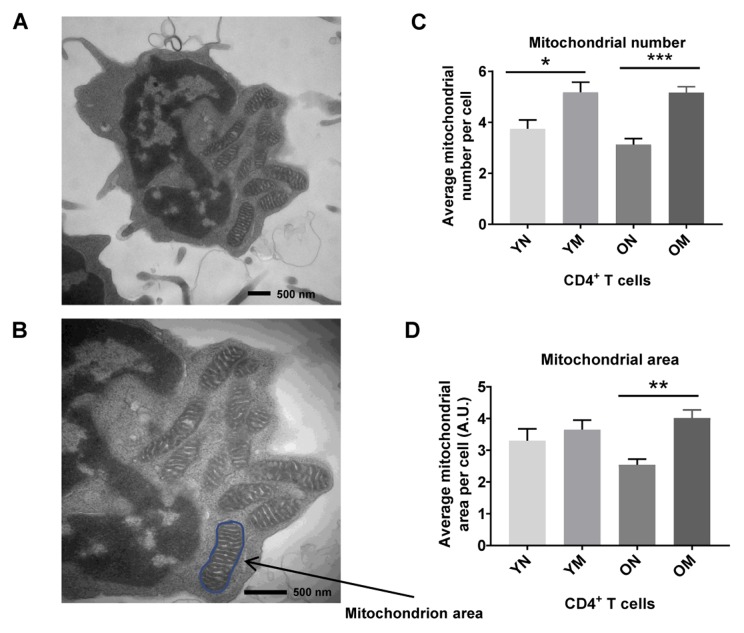
(**A**) TEM image of CD4^+^ T cell showing mitochondria, and (**B**) mitochondrion area (size) outlined in blue. (**C**) Memory (M) CD4^+^ T cells had a significantly higher mitochondria number than naïve (N) cells in cells from both young (Y) and old (O) (*p = 0.0267, ***p = 0.0008, respectively). (**D**) In memory CD4^+^ T cells of both young and old, the mitochondrial area was significantly greater compared to naïve CD4^+^ T cells (**p = 0.0030). Arbitrary Unit (A.U.). (**C**, **D**) *P*-values were calculated by Student’s t-test (two-tailed) using GraphPad PRISM 7 software. Error bars reflect the standard error of the mean (±SEM). N = 5 young, 4 old donors.

We hypothesized that the apparent discrepancy between mitochondrial protein abundance and function reflected the persistence of dysfunctional mitochondria. To gain insight into cause of mitochondrial dysfunction, we visualized mitochondria number, mass (area) and shape in naïve and memory CD4^+^ T lymphocytes from younger (2 men and 3 women; ages 22–34 years-old) and older (4 men; ages 70–80 years-old) participants (Supplementary Table 5) using TEM. In both younger and older participants, memory CD4^+^ T lymphocytes had significantly higher mitochondria counts (*p* = 0.0267, *p* = 0.0008, respectively) than naïve CD4^+^ T lymphocytes (Figure 4C). These observations were consistent with early studies in mouse CD8^+^ memory T cells (32). The counts of structures identified as mitochondria were not significantly different between CD4^+^ naïve or memory T lymphocytes from older and younger participants (Figure 4A–4C). We calculated mitochondrial surface area as a surrogate for mitochondrial mass [32]. In cells from young individuals, average mitochondrial area of naïve and memory CD4^+^ T cells from young individuals were not significantly different (Figure 4D). Conversely, in cells from older individuals, average mitochondrial areas of naïve CD4^+^ T cells were significantly lower than those from CD4^+^ T memory cells (*p* = 0.0030). Though we noted a trend towards lower mitochondrial area in older compared to younger naïve CD4^+^ T cells, such a difference was not statistically significant in this limited cohort.

**Figure 5:**
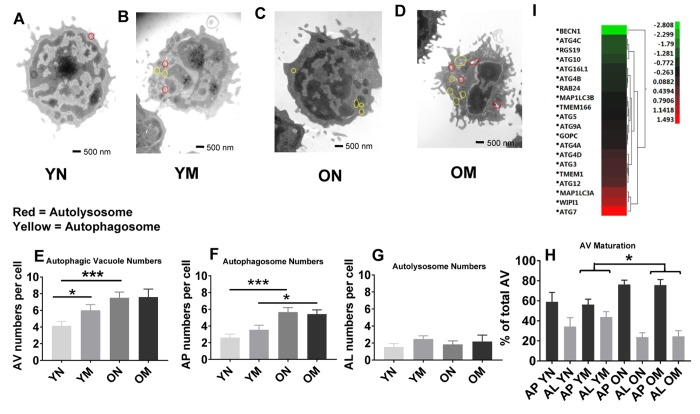
(**A**–**D**) Representative images showing autophagosomes and autolysosomes from young (Y) and old (O) naïve (N) and memory (M) CD4^+^ T cells. Circles in red indicate autolysosomes, in yellow indicate autophagosomes. (**E**) The number of autophagic vacuoles, the combination of autophagosomes and autolysosomes, was increased in naïve CD4^+^ T cells from older as compared to younger individuals (***p = 0.0006) and also increased in memory CD4^+^ T cells from young compared to naïve CD4^+^ T cells from young individuals (*p = 0.0424). (**F**) Autophagosomes were significantly higher in both naïve and memory CD4^+^ T cells from older individuals compared to naïve and memory CD4^+^ T cells of younger individuals (***p = 0.0001, *p = 0.0217, respectively). (**G**) No significant differences were found in the number of autolysosomes from young and old naïve and memory CD4^+^ T cells. (**H**) Significant differences were found in autophagic vacuole (AV) maturation of autophagosomes (AP) transitioning to autolysosomes (AL) between memory CD4^+^ T cells from older individuals compared to younger individuals (*p = 0.022). No differences were found for autophagic vacuole maturation for naïve CD4^+^ T cells. (**E**–**H**) *P*-values were calculated by Student’s t-test (two-tailed) using GraphPad PRISM 7 software. Error bars reflect the standard error of the mean (±SEM). N=5 old, 4 young donors. (**I**) Gene expression data for autophagy pathway genes in CD4^+^ T cells from older compared to young individuals showing up-regulation (above 0-1.5 Z- ratio) of some autophagy-related genes (*ATG7*, *WIPI1*, *MAP1LC3A*, *ATG12*, *TMEM1*, *ATG3*, *ATG4D*). Interestingly, *BECN1* was down-regulated (-2.8 Z-ratio) in older compared to young individuals. N= 8 young, 25 old donors.

To determine if the higher abundance of mitochondrial proteins could be a consequence, at least in part, of altered mitochondrial turnover by autophagy, we carried out morphometric analysis of the two main subcellular compartments related to autophagy, autophagosomes (AP) and autolysosomes (AL), from young and old naïve and memory CD4^+^ T cells. In autophagy, cytosolic components are sequestered inside AP that then fuse with lysosomes (forming AL), where degradation takes place. This process of AP maturation to AL has been shown to be compromised in different cells from old rodents [33]. We set out to analyze possible differences in the total content of autophagic compartments (autophagic vacuoles; AV) and in their state of maturation using TEM and morphometry. For this analysis we used 54 TEM images (15 of naïve cells from 5 young donors, 12 of naïve cells from 4 older donors, 15 of memory cells from 5 young donors, and 12 of memory cells from 4 older donors; Figure 5A–5D and Supplementary Figure 4A, example images). The total count of AV (sum of AP and AL) was significantly higher (*p* = 0.0006, *p* = 0.0001, respectively) in naïve CD4^+^ T lymphocytes subsets from older compared to younger participants. This increase was mostly as result of higher AP abundance (*p* = 0.0001) in naïve CD4^+^ T lymphocytes subsets from older compared to younger participants (Figure 5E, 5F), whereas we found no age-related difference in the number of autolysosomes in both cell types (Figure 5G). These data support differences in AP maturation in CD4^+^ T cells from old individuals. In fact, when we quantified the fraction of AV in the AP or AL state, we found that both memory and naïve CD4^+^ T cells from older individuals displayed higher percentage of AV in an immature (AP) state compared to younger individuals (Figure 5H). These differences reached statistical significance in memory CD4^+^ T cells from older individuals (*p* = 0.022) where clusters of AP were a common occurrence (examples in Supplementary Figure 4A panels a, c and d). AP-occupied areas expressed in absolute terms or as percentage of total cytosol area were significantly higher (*p* = 0.0005, *p* = 0.0002, respectively) in older than younger participants in naïve CD4^+^ T cells but not in memory CD4^+^ T cells (Supplementary Figure 4E and 4H). In further support that the increase in AV was mostly due to reduced maturation/clearance rather than increased AP biogenesis, transcriptome analysis indicated that expression of most autophagy genes was not different in CD4^+^ T cells from both age groups, and Beclin-1 (*BECN1*), that participates in autophagy induction, was substantially underrepresented in CD4^+^ T lymphocytes from older compared to younger participants (Figure 5I). Only *ATG7* was overrepresented in the older group, but whether this is a compensatory reaction or related with some of the non-autophagy related functions of *ATG7* recently described [34], will require future investigation.

Overall, the TEM data showed in CD4^+^ naïve and memory T lymphocytes from older participants the permanence of dysmorphic mitochondria and higher abundance of undegraded immature AP. In many instances, mitochondria were observed inside the AP that accumulated in CD4^+^ T cells from older patients (Supplementary Figure 4A panels b–e), suggesting that part of the altered mitochondria were sequestered from the cytosol but failed to undergo degradation. These findings could explain why the higher content of mitochondrial proteins in older patient cells did not associate with higher number or size of mitochondria.

### Mitophagy detection in CD4^+^ T cells

**Figure 6:**
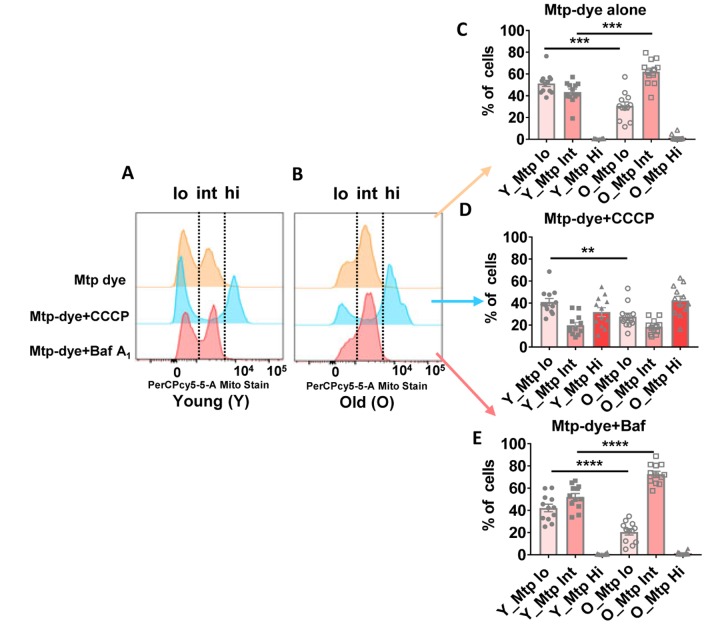
(**A** and **B**) Representative histograms from a young and old donor for all conditions using Mtphagy Dye (20,000 cells recorded). Summary data showing percentage of CD4^+^ T cells of young and old donors divided by fluorescent intensity (low [‘lo’], intermediate (‘int’), and high [‘hi’]) of Mtphagy Dye using, © Mtphagy Dye alone, (**D**) CCCP administration, and (**E**) Bafilomycin A_1_ administration. ****p = 0.0001, ***p = 0.0002 and 0.0003, **p = 0.0081. (**C**–**E**) *P*-values were calculated by Student’s t-test (two-tailed) using GraphPad PRISM 7 software. Error bars reflect the standard error of the mean (±SEM). N = 12 young, 12 old donors.

To better understand the basis for morphological differences between mitochondria present in CD4^+^ T cells from older compared to younger individuals, we assessed mitophagy flux using flow cytometry and a mitochondria-specific fluorescent dye whose emission intensity increases as organelle pH acidifies [35]. Using this method, mitochondria in cytoplasm can be distinguished from those within immature autolysosomes and autolysosomes because of their lower pHs [36]. We incubated CD4^+^ T cells from 24 donors (Supplementary Table 6) ranging in age from 23–90 years-old with Mtphagy Dye for 30 minutes, and then further cultured the cells for 18 hours with no added pharmacologic agents. We found that a greater proportion of mitochondria from older individuals had intermediate fluorescence intensity compared to cells from younger individuals (Figure 6A, 6B, top row marked ‘int’ for a representative younger and older individual [see Supplementary Figure 5A, 5B for dot plots for a representative younger and older individual]; quantified for all individuals in Figure 6C), supporting that mitochondria were healthier in the younger individuals and that, at a given time, the amount of mitochondria requiring to undergo mitophagy and already trapped in autophagic compartments was higher in cells from older individuals. To compare mitophagy flux in both groups of individuals, we forced mitochondrial degradation by culturing the cells for 18 hours in the presence of Carbonyl cyanide m-chlorophenyl hydrazone (CCCP, a mitochondrial transport uncoupler [37]), and found that this agent was still able to efficiently drive mitochondria into a high fluorescent intensity (low pH) compartment in CD4^+^ T cells from older donors (Figure 6A, 6B, middle row marked ‘hi’ for a representative younger and older individual; quantified for all individuals in Figure 6D). The results after adding CCCP support that cargo-recognition and mitophagy induction were still preserved in CD4^+^ T cells from older donors, and that as in the case of the autophagic flux analysis, the higher presence of mitochondria in intermediate pH compartments in the older group was a consequence of impaired maturation/degradation. We confirmed that bafilomycin A_1_ treatment, which prevents vacuolar acidification [38], increased the fraction of dye in the intermediate fluorescence (immature autophagic compartments) in both groups of age (quantified for all individuals in Figure 6E), but differences between CD4^+^ T cells from young and older individuals were still noticeable, in support that defective clearance of autophagic compartments in the older group may not be due to differences in acidification but rather in cargo degradation. We infer that a greater proportion of mitochondria in CD4^+^ T cells from the elderly are already ‘stuck’ in autophagic compartments due to poor clearance in these compartments.

## DISCUSSION

The aim of this study was to gain understanding of the mechanisms that in old age lead to immunosenescence. In spite of extensive research in this area, the nature of these mechanisms is still unknown [39]. We started by performing a proteomic discovery study that compared cytoplasmic extracts from CD4^+^ T lymphocytes obtained from younger and older healthy participants of the BLSA. We focused on CD4^+^ T lymphocytes because in previous studies we had found that aging is associated with profound changes in the basal transcriptional profile of CD4^+^ T lymphocyte toward NF-κB activation and a proinflammatory state [29, 30].

Differently from previous studies, we performed a discovery proteomic analysis on cytoplasmic extract because our prior findings suggested that the origin of the proinflammatory state of aging in CD4^+^ T lymphocytes was metabolic in nature [30]. We found that mitochondrial proteins were overrepresented in the cytoplasm of CD4^+^ T lymphocytes from older compared to younger participants while gene expression analyses showed opposite trends with mitochondrial transcripts underrepresented in CD4^+^ T cells from older compared to younger individuals. We hypothesized that these findings could be explained by the persistence of damaged and dysfunctional mitochondria that were not recycled. In keeping with this hypothesis, CD4^+^ T cells from older compared to younger participants had impaired mitochondrial respiration; TEM images as well as flow cytometric studies further supported the possibility of more mitochondria enclosed in autophagic compartments due to impaired autophagic flux in cells from older donors. We infer that dysfunctional mitochondria remained unrecycled in cells because of defective autophagy at the level of clearance of the already sequestered mitochondria. We also noted a trend towards reduced mitochondrial mass in naïve CD4^+^ T cells in older individuals, whereas no differences were noted in memory CD4^+^ T cells. These observations point to the possibility that 1) older memory CD4^+^ T cells are being generated from residual functional naïve CD4^+^ T cells in the elderly, or 2) old memory CD4^+^ T cells were generated early and have survived as the individual aged.

Processes that are seen as seminal components of aging across systems [9, 40], such as the loss of proteostasis and mitochondrial dysfunction, may hold the key to deciphering immune cell-specific effects of aging [11]. A chief regulator of proteostasis, autophagy plays an important role in the balance between protein synthesis and degradation [41]. Mitochondrial dysfunction may curtail energy availability and further impair mitochondrial turnover as well as biogenesis [9]. The balance between biogenesis and mitophagy is particularly important in long-lived cells such as neurons, cardiac cells, and memory T cells [9, 41].

Our proteomic findings cannot be directly compared with the current the literature because, as far as we could find, no previous study performed quantitative discovery proteomics in cytoplasmic extracts from human CD4^+^ T cells and compared younger and older healthy individuals. While the decline of autophagic capacity with age has been previously noted, our studies directly implicate this mechanism in human immunosenescence and suggest a plausible cause for elevated basal NF-κB dependent gene expression, and associated mild proinflammatory state in these cells [42]. The crux of our model is that decreased capacity to clear dysfunctional mitochondria through autophagy with age and contributes to increased oxidative stress [43]. ROS are well known activators of NF-κB which could lead to increased NF-κB gene transcription noted in our gene expression studies [29, 30]. Additionally, accumulation of damaged mitochondria creates an energetic deficit, and depolarized mitochondria release mitochondrial Damage-Associated Molecular Patterns, such as mitochondrial DNA, cardiolipin, ROS, and other compounds that contribute to NLRP3 inflammasome activation. [44]. We hypothesize that simultaneous NF-κB dependent gene expression and inflammasome activation in resting CD4^+^ T cells contribute to the low-grade proinflammatory state of aging.

It has been clearly established that increased mitochondrial mass and related expansion of respiratory capacity and ATP production is an essential prerequisite for memory lymphocytes development and effective proliferation upon secondary exposure to antigens [45]. Of note, the activation of the NLRP3 inflammasome is also facilitated by increased NF-κB signaling in CD4^+^ T lymphocytes from older compared to younger individuals [30]. This observation is also consistent with previous evidence that NF-κB up-regulation in older persons is cell-intrinsic and mediated in part by phosphatidylinositol 3-kinase (PI3K) activity induced in response to metabolic activity, which can be moderated by rapamycin treatment. These data suggest that inhibition of the mTORC signaling may reverse, at least in part, some of the effects of immunosenescence [30]. This is consistent with the notion that fasting mimicking diets and other dietary interventions that affect mTOR signaling and enhances autophagy positively affect immune function [46].

A lingering question that arises from our findings is why depolarized mitochondria do not precipitate apoptosis through the release of cytochrome C and activation of caspase-9 [47]. Indeed, human aging has been associated with increased apoptosis in the T cell compartment [48]. Our data indicate that mitochondria recognition and sequestration inside autophagosomes is still normal in CD4^+^ T cells from older individuals and therefore even if cytochrome C is released it would already be inside autophagosomes where it would not activate apoptosis. This would suggest that the apoptosis observed in the T cell compartment in human aging may not originate from depolarized mitochondrial signaling but perhaps originates from other signaling places. We have also found Bcl2 to be higher in older cells which may contribute to reduced apoptosis.

Our findings are also somewhat consistent with Macian and collaborators who showed that T cells of older individuals who are the progeny of non-centenarians show reduced autophagic flux that is correlated to a loss of immune function when compared to T cells of older offspring of centenarians and other individuals of exceptional longevity [14]. Finally, our findings are consistent with the recent work of Reynolds and collaborators who performed transcriptomic profiling in CD14^+^ monocytes in participants of the Multi-Ethnic Study of Atherosclerosis (ages 55–94 years-old) and found both reduced gene expression for genes coding for mitochondrial proteins, in particular proteins related to oxidative phosphorylation, and also significant dysregulation of transcripts coding for proteins related to autophagy [49].

Our findings have important implications for treatment and prevention of immunosenescence and its consequences. Previous research demonstrated that aging-induced changes in the subpopulations of effector T cells and regulatory CD4^+^ T lymphocytes is associated with poor responses to influenza vaccination [50]. A number of recent studies have found that the mTOR inhibitor RAD001, an analog of rapamycin, improves immune function in older humans. Pretreatment with low doses of RAD001 improves the response to influenza vaccination [26], although effects of rapamycin analogs on immune parameters have not been confirmed in older populations [51]. Nevertheless, our findings suggest that targeting autophagy may be an effective approach to slow down immunosenescence and prevents its deleterious consequences, such as “inflammaging” and blunted immune response to pathogens and vaccination.

Our study has unique features that enhance the value and robustness of our findings. All the experiments were conducted on CD4^+^ T lymphocytes immediately after their collection and isolation from BLSA participants who are selected to be very healthy. Therefore, our findings are not influenced by clinically evident pathology in older individuals or by sample storage. However, our study also has limitations. For example, the proteomic experiment was performed in total CD4^+^ T lymphocytes, without distinction between naïve and memory subclass. This choice was induced by the need to having enough cells to obtain enough cytoplastic extract to perform the proteomic study. However, previous research has shown that the proportion of naïve/memory do not significantly change with aging in CD4^+^ T lymphocytes while it dramatically changes in CD8^+^ T lymphocytes [28]. It is also possible that the conditions and times required to purify cells by flow cytometry may alter basal gene expression and respiratory patterns. The effects of flow cytometry on these sensitive biochemical parameters need to be rigorously evaluated in future studies. Additionally, the proteomics study was coordinated only with male donors, further studies with female donors will be needed to identify sex-specific differences in the proteomes of CD4^+^ T lymphocytes. We note, however, that gene expression profiles identified many of the same mitochondria related pathways being affected by age regardless of sex. Another limitation is that the different experiments were conducted on cells collected from different BLSA participants. Given the strict inclusion criteria for participation in this study, the heterogeneity of results is probably modest, but we cannot exclude that some significant variation was introduced. Finally, while we identified impaired autophagy and mitochondria chronic damage as possible factors affecting immunosenescence, we did not quantify these parameters in a larger population correlated with some of the traditional signs of immune dysfunction or studied under condition of lymphocyte activation. Future studies should be designed to address this limitation and gain further insight into the effect of aging on CD4^+^ T cell function.

In conclusion, we found evidence of defective mitochondrial recycling in CD4^+^ T lymphocytes from older compared to younger persons and some suggestion that the lack of mitochondrial recycling can be attributed to defective autophagy. If the functional relevance of these findings can be confirmed in future studies, interventions that enhance autophagy should be tested as strategies to prevent or slow down the progression of immunosenescence.

## MATERIALS AND METHODS

### Study populations

Samples were derived either from BLSA participants or normal donors. Participants enrolled in the BLSA (NIA protocol #03-AG-0325) are volunteers who at the time of study enrollment were “healthy” based on strict eligibility criteria. BLSA participants tend to be healthier than the general population. Young normal donors are volunteers who have donated blood through the Tissue Procurement for Biomedical Research protocol (NIA protocol #03-AG-N322) or apheresis material through the Cytapheresis of Volunteer Donors protocol (NIA protocol #03-AG-N316). All individuals were consented for their donations and protocols have been reviewed by the appropriate institutional review board at NIH.

### Proteomics

### Subjects

Twenty-two men (21–83 years-old) were enrolled. Samples were taken from apheresis material from older donors from the BLSA and younger normal donors through the NIA Cytapheresis of Volunteer Donors protocol. This study required a large quantity of WBCs that could only be obtained by apheresis.

### Cell preparation

PBMCs were isolated from apheresis packs using Ficoll-Paque Plus (GE Healthcare, Piscataway, NJ, USA) density gradient. CD4^+^ T cells were obtained by positive selection using anti-human CD4 microbeads (Miltenyi Biotec, Auburn, CA, USA). Cells were kept overnight at 4 °C.

### Protein extraction

6×10^7^ CD4^+^ T cells in 18 mL culture medium were plated. Cells were rested overnight at 4 °C in RPMI 1640 + 1% penicillin/streptomycin, 1% L-glutamine (P/S/G) + 10% fetal bovine serum (FBS) medium. Cells were then incubated for 4 hours in 5% CO2 at 37 °C. Cells were collected by centrifugation and used to prepare cytoplasmic extracts (CE) [29, 30]. CE proteins were quantified using the Bradford protein assay.

### Protein digestion and labeling with iTRAQ reagent

Briefly, 50 μg of each extract were mixed with six volumes of cold acetone (−20 °C) for overnight precipitation. The samples were centrifuged at 4°C, acetone was removed, and pellets were air dried. The pellets were then resuspended in dissolution buffer (0.5 M triethylammonium bicarbonate [TEAB], 0.2% SDS), then reduced and alkylated before digesting overnight at 37 °C using sequencing-grade modified trypsin (Promega, Maddison, WI) at a protein-to-enzyme ratio of 20:1 according to iTRAQ 8plex labeling protocol (ABSciex, Foster city, CA). Using the iTRAQ kit (ABSciex, Foster City, CA), protocol peptides were then labeled with iTRAQ tags as seen in Supplementary Table 1. One common reference sample was used in all four different iTRAQ 8plex experiments, which would help in normalization among iTRAQ runs and also to compare samples from one iTRAQ to another. We also used a technical control sample within an iTRAQ run and used three technical control samples between iTRAQ runs. After stopping the labeling reaction, the resulting labeled peptides were all combined and dried. The samples were then resuspended in 0.1% formic acid to a final volume of 1 mL and then desalted using Waters Oasis HLB 1cm cartridges (Milford, MA) per the manufacturer’s instruction. The eluate was then dried and resuspended in reverse phase solvent buffer A (10 mM TEAB in water, pH 8.4-8.6). The combined peptide digest was fractionated offline by basic reverse phase fractionation followed by online reverse phase liquid chromatography-tandem mass spectrometry.

### Peptide fractionation using basic reverse phase chromatography and LC-MS/MS analysis

The pooled mixture of iTRAQ-labeled peptides was fractionated using basic reverse phase chromatography on an Agilent 1200 HPLC using a XBridge C18 Column, 5µm, 2.1x100 mm (Waters, Milford, MA). A 60 min linear gradient from 0% to 40% Buffer B (10 mM TEAB in 90% Acetonitrile, pH 8.4-8.6)) was used to separate the peptides with a flow rate of 200 µL/min with a total run time of 90 minutes. Fractions were collected at 1-minute intervals on a 96-well microplate with a total of 90 fractions. The fractions were then pooled to a total of 12 fractions. Each fraction was completely dried using a SpeedVac (Thermo Fisher Scientific, San Jose, CA) and then resuspended in 0.1% formic acid before injection for LC-MS/MS analysis. Fractions were collected at 1-minute intervals on a 96-well microplate with a total of 90 fractions. The fractions were then pooled to a total of 12 fractions. Each fraction was completely dried using a SpeedVac and then resuspended in 0.1% formic acid before injection for LC-MS/MS analysis. Liquid chromatography-tandem mass spectrometry was performed using the Eksigent nanoLC-Ultra 1D plus system (Dublin, CA) coupled to an LTQ Orbitrap Elite mass spectrometer (Thermo Fisher Scientific, San Jose, CA). Ten µL of peptide digest was first loaded onto a Zorbax 300SB-C18 trap column (Agilent, Palo Alto, CA) and then separated on a reverse-phase PicoFrit analytical column (New Objective, Woburn, MA) using a 40-min linear gradient of 5–40% acetonitrile in 0.1% formic acid with a flow rate of 250 nL/min. LTQ-Orbitrap Elite settings were as follows: spray voltage 1.5kV; full MS range: m/z 300-2000, data-dependent acquisition. A full MS-scan was followed by six data-dependent MS2 scans for precursor ions.

### Protein identification/data analysis

Protein identification and quantitation using the MS/MS spectra was performed using the Proteome Discoverer v1.3 software (Thermo Fisher Scientific, San Jose, CA) using Mascot search engine (Matrix Science, Boston, MA). The search criteria were set to the following: database, Swiss-Prot (Swiss Institute of Bioinformatics); taxonomy, human; enzyme, trypsin; miscleavages, 3; variable modifications, oxidation (M), deamidation (NQ), iTRAQ 8plex tyrosine; fixed modifications, (MMTS) methyl methanethiosulfonate ©, N-terminal iTRAQ 8plex, iTRAQ 8plex lysine, MS peptide tolerance, 10 ppm; MS/MS tolerance, 0.05 Da. Proteins where fold changes in protein expression were greater than 1.3 or less than 0.7 were examined.

### Statistical analysis

Data were analyzed within each iTRAQ 8plex and also with all combined. Differences between young and old were determined by Student’s t-test (two-tailed) and proteins that met the following criteria were ranked by significance (only *p* < 0.05 shown). Proteins were selected that were up- or down-regulated (ratio >1.3 or <0.7 and had a *p* < 0.05). Averages of ratios were calculated between old and young (averages [old] / averages [young]) after normalization using the common control sample used across iTRAQ 8plex experiments. Two hundred and twenty-four proteins had ratios of 1.3 or greater and 56 proteins had ratios of <0.7 or smaller. Proteins were then selected according their *p*-value (*p* < 0.05). One hundred and four proteins fitted the criteria indicated above out of 3,510 proteins after this three-step analysis. Twenty-nine of these 104 proteins were determined to be significantly up-regulated in the old after accounting for unique peptides (≥ 2, similar peptides sequences not counted), high confidence of the peptide sequences (medium and low confidence peptide sequences eliminated), and ion score of more than 30. DAVID v.6.7 (bioinformatics tool) was then used to convert between protein identifiers/gene. The mass spectrometry proteomics data have been deposited to the ProteomeXchange Consortium via the PRIDE partner repository with the dataset identifier PXD016039. .

### Gene expression

Thirty-three donors (24 men and 9 women) were enrolled, age range of 25–82 years-old (Supplementary Table 3). All blood samples were taken from older donors from the BLSA and one younger normal donor through the NIA Cytapheresis of Volunteer Donors protocol.

### Cell preparation

PBMCs were isolated from apheresis packs using Ficoll-Paque Plus (GE Healthcare, Piscataway, NJ, USA) density gradient. CD4^+^ T cells were obtained by positive selection using anti-human CD4 microbeads (Miltenyi Biotec Auburn, CA, USA) [29]. CD4^+^ T cells in RPMI 1640+1% P/S/G+10% FBS medium were incubated for 4 hours at 37ºC (5% CO_2_ incubator). CD4^+^ T cells were then washed and collected by centrifugation. Cell pellets were then lysed in 350 μl RLT buffer (QIAGEN Inc, Valencia, CA, USA) for total RNA extraction [29, 30].

### Total RNA purification and microarray hybridization

Total RNA was extracted from frozen cell pellets containing 5×10^6^ cells using the Qiagen RNeasy Mini Kit (QIAGEN Inc, Valencia, CA, USA) as previously described [29].

### Microarray hybridizations

Total RNA was used to generate biotin-labeled single-strand RNA (cRNA) using the Illumina Total Prep RNA Amplification Kit (Ambion, Austin, TX, USA) according to the manufacturer's recommendations as previously described [30]. 0.75 μg of biotin-labeled cRNA was hybridized for 16 hours at 58^o^C to Illumina's Sentrix HumanRef-8 Expression BeadChips (Illumina, San Diego, CA, USA). The arrays were washed and blocked, and then the labeled cRNA was detected by streptavidin-Cy3 staining. Arrays were scanned using an Illumina BeadStation 500X Genetic Analysis Systems scanner and the image data extracted using Illumina BeadStudio software, version 3.0 [29].

### Microarray data analysis

Microarray data were analyzed using DIANE 6.0 as previously described [29, 30]. Raw microarray data were subjected to filtering by the detection *p*-values and then normalized by Z-transformation with log signal values. The data were further tested for significant changes as previously described [29]. Individual genes with Z-ratio ≥ 1.5, *p*-value ≤ 0.05 and false discovery rate (FDR) ≤ 0.3 were considered significantly changed. The PAGE algorithm was employed for gene set enrichment analysis by using all the genes in each sample as input against and the data set supplied by Gene Ontology Institute and pathway gene set of the MIT Broad Institute molecular signature database. For each relevant comparison, the lists of differentially expressed genes and Z-ratios were entered into the PAGE Pathway Analysis software to organize them according to known biological pathways. The enrichment Z-scores for each functional grouping were calculated based on the chance of mRNA abundance changes predicting these interactions and networks by Z-test. Pathways *p*- ≤ 0.05 and pathways FDR ≤ 0.3 were the cutoff criteria for significant pathway selection. Up and down top 50 pathway Z-scores were averaged amongst young (Y) (20-30) and old (O) (70-80) donors, and Z-score differences between young and old are shown on the X-axis. Each row denoted a different pathway (*p* ≤ 0.05 and FDR ≤ 0.3).

### Accession number

The microarray GEO accession numbers for the data reported in this paper is GSE131407.

### Mitochondrial respiration

### Subjects

Fourteen Caucasian men (23–82 years-old) were enrolled for this study. All blood samples were taken from old donors selected from the BLSA and younger donors selected from the NIA Tissue Procurement for Biomedical Research protocol. All participants were fasting at time of blood draw.

### Cell preparation

PBMCs were isolated from 5 of 10 mL heparinized whole blood tubes. CD4^**+**^ T cells were isolated from PBMCs using the immunomagnetic negative selection kit (EasySep Human CD4^**+**^ T cells Enrichment Kit; StemCell Technologies, Vancouver, BC, Canada) using RoboSep, a fully automated cell separator (StemCell Technologies) according to the manufacturer's protocol.

### Flow cytometry

CD4^+^ T cells were stained using the following fluorescent antibodies: CD4 (FITC), CD45RA (APC), CD62L (PECy7) from BD Bioscience. Cells were then analyzed by flow cytometry (BD FACSCANTO II). Data were analyzed to define % of naïve, central memory (CM), effector memory (EM) CD4^+^ T cell percentages using FLOWJO software (version 10).

### Mitochondrial respiration

Mitochondrial respiration was measured using high-resolution respirometry (Oxygraph-2 k; Oroboros Instruments, Innsbruck, Austria) [31]. The O2k is a 2-chamber high resolution respirometer for monitoring oxygen consumption from small amounts of biological samples. A suspension of CD4^+^ T cells in culture medium (RPMI 1640 medium, 10% FBC, 1% P/S/G) was added to the Oxygraph-2k chambers at a concentration of 5×10^6^ cells in 2 mL. After 10 minutes of stabilization at 37 °C, the coupling control protocol was applied to evaluate the cellular routine respiratory state, the mitochondrial coupling state, non-coupled respiratory capacity, and rotenone-insensitive or residual oxygen consumption [52]. Briefly, Oligomycin (2 μg/mL) was added to inhibit ATP synthase and induce non-phosphorylating (LEAK) respiration (electron flow coupled to proton pumping to compensate for proton leaks). Maximal respiratory capacity of the electron transfer system was subsequently obtained by titrating the uncoupler FCCP (injected stepwise up to 1–1.5 μM). Finally, rotenone (0.5 μM) and antimycin-A (2.5 mM) were added to measure the non-coupled respiratory capacity, and rotenone-insensitive or residual oxygen consumption.

### Bioenergetic health index (BHI)

The BHI, a summary index of mitochondrial function in cells [31] was calculated for each sample. The BHI is derived from calculating a ratio of positive aspects of mitochondrial bioenergetic function (i.e. reserve capacity and ATP-linked respiration) to potentially deleterious aspects of mitochondrial bioenergetic function (i.e. non-mitochondrial oxygen consumption and proton leak) as follows:

$$\text{BHI}=\log=\frac{\left(\text{reserve}\;\text{capacity}\right)\times \left(\text{ATP-linked}\right)}{\left(\text{non-mitochondrial}\right)\times \left(\text{proton leak}\right)}$$

### Transmission Electron Microscopy (TEM)

Nine donors (6 men and 3 women) were analyzed for this study, age range 22–80 years-old. Older subjects for this study were selected from the BLSA, younger donors were from the NIA Tissue Procurement for Biomedical Research protocol.

### Cell preparation

PBMCs were isolated from 50 mL apheresis heparinized blood using Ficoll-Paque Plus (GE Healthcare, Piscataway, NJ, USA) density gradient.

### CD4^+^ T cells

CD4^**+**^ T cells were isolated from PBMCs using the immunomagnetic negative selection kit (EasySep Human CD4^**+**^ T cells Enrichment Kit; StemCell Technologies, Vancouver, BC, Canada) using a fully automated cell separator (RoboSep, StemCell Technologies) according to the manufacturer's protocol.

### Flow cytometry

CD4^+^ T cell were stained using the following fluorescent antibodies: CD4 (FITC), CD45RA (APC), CD62L (PECy7) from BD Bioscience, then cells were sorted to naïve and memory cells using a BD FACSAria flow cytometer.

### TEM

Five to seven million naïve and also 5–7 × 10^6^ memory CD4^+^ T cells were fixed in 5% glutaraldehyde (GA), 6 mM MgCl_2,_ in 0.2 M sodium cacodylate buffer for 10 minutes at 4 °C then kept in 2.5% GA, 3 mM MgCl_2,_ in 0.1 M sodium cacodylate buffer (pH 7.2-7.4) under continuous motion (rotary mixer) overnight at 4°C.

After buffer rinse, samples were postfixed in 1% osmium tetroxide, 0.8% potassium ferrocyanide in 0.1 M sodium cacodylate buffer (1 hour) on ice in the dark. Following a DH_2_O rinse, plates were stained with 2% aqueous uranyl acetate (0.22 μm filtered, 1 hour in the dark), dehydrated in a graded series of ethanol, and embedded in Eponate 12 (Ted Pella) resin. Samples were polymerized at 60°C overnight.

Thin sections, 60 to 90 nm, were cut with a diamond knife on the Reichert-Jung Ultracut E ultramicrotome and picked up with 2×1 mm formvar coated copper slot grids. Grids were stained with 1% tannic acid, 2% uranyl acetate in 50% methanol and 0.4% lead citrate before imaging on a Philips CM120 at 80 kV. Images were captured with an AMT XR80 CCD (8 megapixel) camera.

### Images

Twenty to 40 cell images were taken per sample. For each cell, we obtained whole cell images (direct magnification 17,500×) and also partial cell images of mitochondrial areas (one to three depending on mitochondrial areas; Direct Mag 33,000×). We processed 368 cell images and counted 3,059 mitochondria. We traced the perimeter of each mitochondrion to find out the area (size). We analyzed 54 images for morphometric analysis.

### Morphometric analysis

AV were identified using previously established criteria [53, 54]. AV (vesicles, 0.5 mm) were classified as AP when they met two or more of the following criteria: double membranes (complete or at partially visible), absence of ribosomes attached to the membrane’s cytosolic side, luminal density similar to cytosol, and/or identifiable organelles or regions of organelles in their lumen. Vesicles of similar size but with a single membrane (or less than 40% of the membrane having a double portion), luminal density lower than the surrounding cytosol, or multiple single membrane-limited vesicles containing light or dense amorphous material were classified as AL. Total AV were composed of the sum of AP and AL. Maturation of AV was calculated as the percent of AL making up AV.

### Mitophagy detection in CD4^+^ T cells

### Subjects

Twenty-four donors (12 men [6 Caucasian and 6 African American] and 12 women [6 Caucasian and 6 African American]) were enrolled for this study. Blood samples were donated from “healthy’ volunteers selected from the BLSA and Tissue Procurement for Biomedical Research protocols. All blood draws were from fasting participants.

### Cell preparation

PBMCs were isolated from four 10 mL heparinized whole blood tubes. CD4^+^ T cells were isolated from PBMCs using the CD4^+^ negative selection kit (StemCell Technologies, Vancouver, Canada) and RoboSep (StemCell Technologies) according to the manufacturer's protocol. Resting CD4^+^ T cells in 10 mL RPMI 1640 complete medium (10% FBS and 1% P/S/G) were incubated for 2 hours at 37°C. Cells were divided into 0.5×10^6^ cells in 0.5 mL 1640 media for four different conditions for mitophagy detection.

### Mitophagy detection

Mitophagy detection experiments were performed using the Mitophagy Detection Kit (Dojindo Molecular Technologies, Inc, Rockville, MD, USA) for live cells using manufacturer's instruction with small modifications. Briefly, 2×10^6^ CD4^+^ T cells were divided into four tubes containing serum-free RPMI. Tube #1 was for unstained cells that followed all washing and media changing processes. 100 nmol/l Mtphagy Dye working solution was added to tubes #2, 3, 4 and then all tubes were incubated at 37°C for 30 minutes. The cells were then washed with serum-free medium. After discarding the supernatant, complete medium (RPMI 1640, 10% FBS, 1% P/S/G) was added to all the tubes. Ten μmol/l CCCP (Sigma-Aldrich) mitophagy-inducer was then added to tube #3, 100 nmol/l Bafilomycin A_1_ (Sigma-Aldrich) autophagy inhibitor was added to tube #4. All tubes were then incubated at 37 °C for 18 hours. After 18 hours, cells were washed with FACS buffer. Mtphagy Dye fluorescence detection by flow cytometry (BD FACSCANTO II) was performed using 488 nm for excitation and 695 nm for emission (this corresponds to the PerCP cy5.5 channel). Data were analyzed using FLOWJO software (version 10).

The Mitophagy Detection Kit was designed for easy use for adhesive cells such as HeLa cells to detect in live cell imaging of mitochondrial autophagy using confocal microscopy. Since CD4^+^ T cells are primary suspension cells, tubes were used instead of the plates for fluorescence detection by flow cytometry. Some images were taken using confocal microscopy to examine the intensity (weak and strong) of the Mtphagy Dye for mitophagy detection but the quantification experiments were performed using flow cytometry (BD FACSCANTO II) and analyzed using FLOWJO V10 software.

## Supplementary Material

Supplementary Figures

Supplementary Tables
