# Supplementary material for: Age-associated changes in human CD4+ T cells point to mitochondrial dysfunction consequent to impaired autophagy
Source: Aging (Albany NY). 2019 Nov 9;11(21):9234–63. doi: 10.18632/aging.102438 (PMC6874450; doi:10.18632/aging.102438)
Supplement: Supplementary Figures [file aging-11-102438-s002.pdf]

SUPPLEMENTARY FIGURES

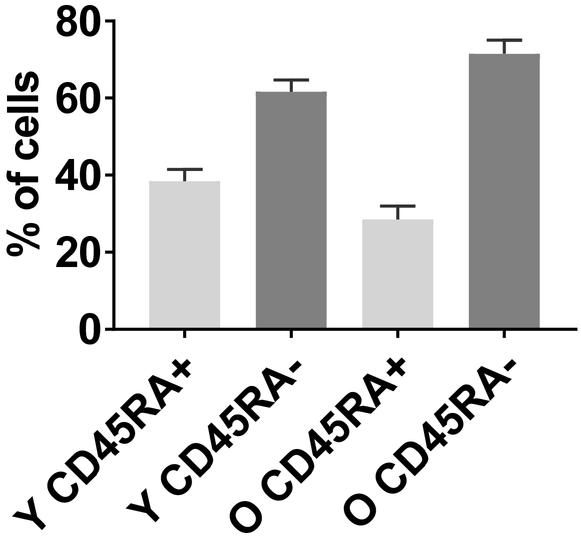

Supplementary Figure 1. FACS profile of $CD4^{+}$T cells in iTRAQ samples. The percentage of naïve $CD4^{+}$T cells analyzed based on $CD45RA^{+}$ expression by flow cytometry. The expression of $CD45RA^{+}$ in older (O) donors 70-83 years-old versus younger (Y) donors 21–34 years-old was comparable (*p*=0.1540) in the iTRAQ experiment sample groups. *P*-value was calculated by Student's t-test using GraphPad PRISM 7 software. Error bars reflect the standard error of the mean (±SEM). N = 4 young,18 old donors..

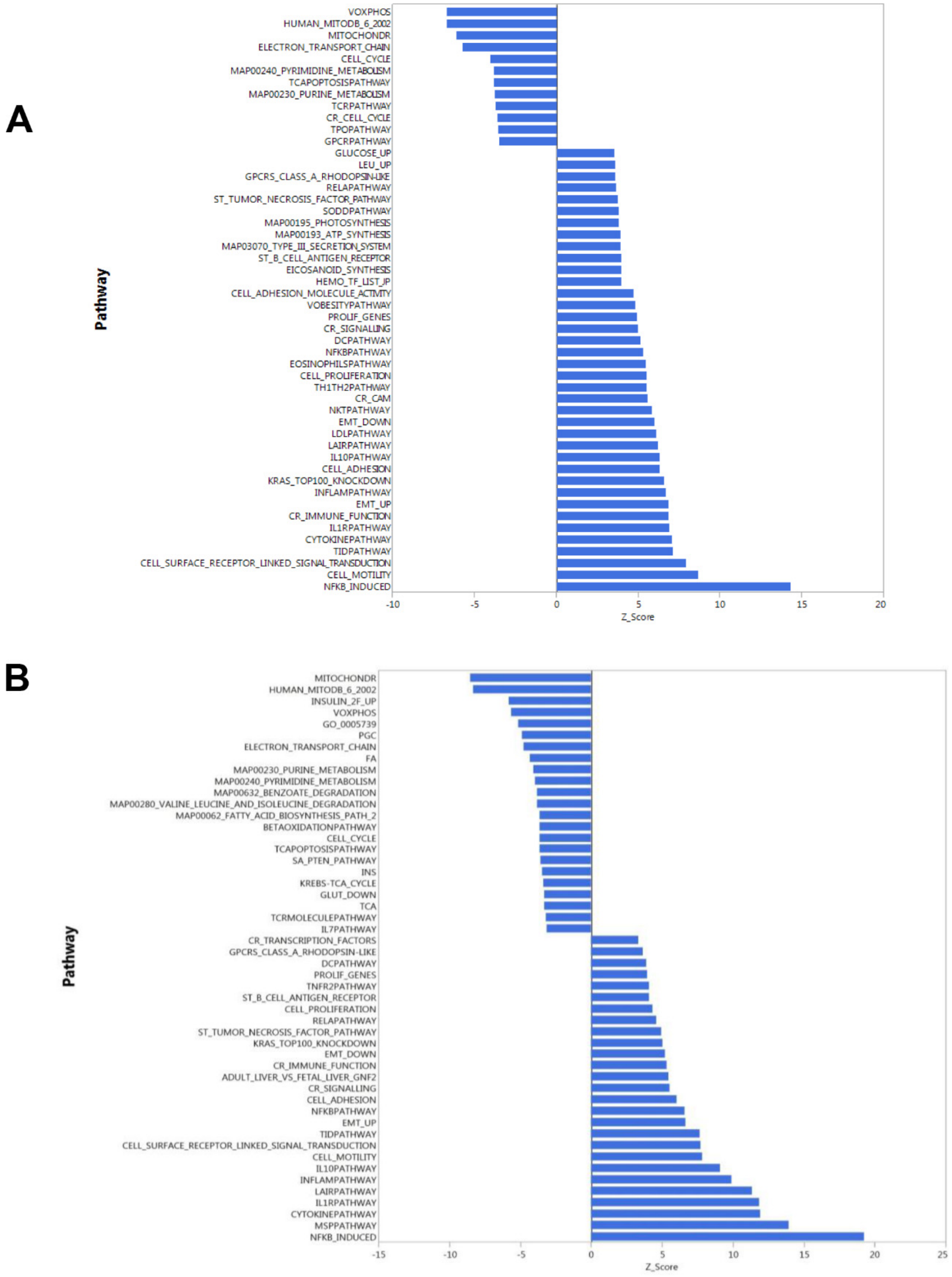

Supplementary Figure 2. (**A**) Gene expression analysis showing top 50 up-and down-regulated biological pathways in CD4$^+$T cells of older (70 to 80 years old) as compared to younger (20 to 30 years-old) women. N = 3 young, 6 old donors. (**B**) Gene expression analysis showing top 50 up-and down-regulated biological pathways in CD4$^+$T cells of older (70 to 80 years-old) compared to younger (20 to 30 years-old) women and men. N = 8 young, 25 old donors.

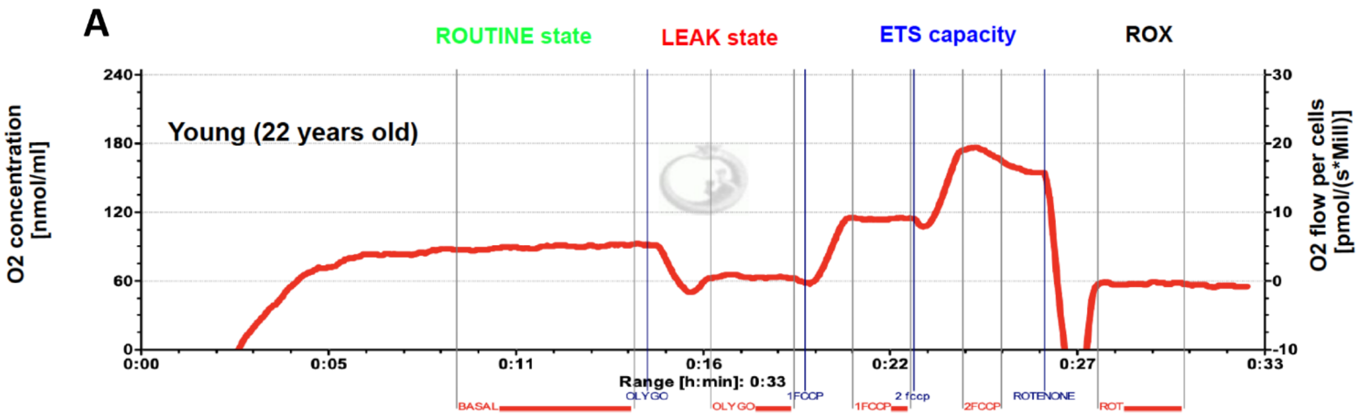

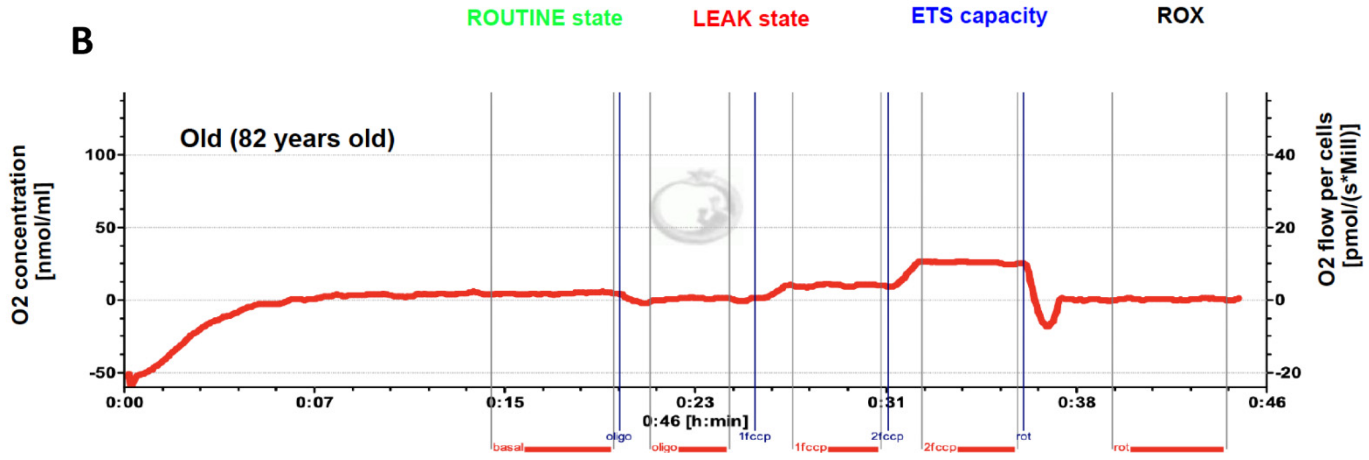

Supplementary Figure 3. Representative mitochondrial respiration measurements using OROBOROS from a young (**A**) and an old (**B**) donor.

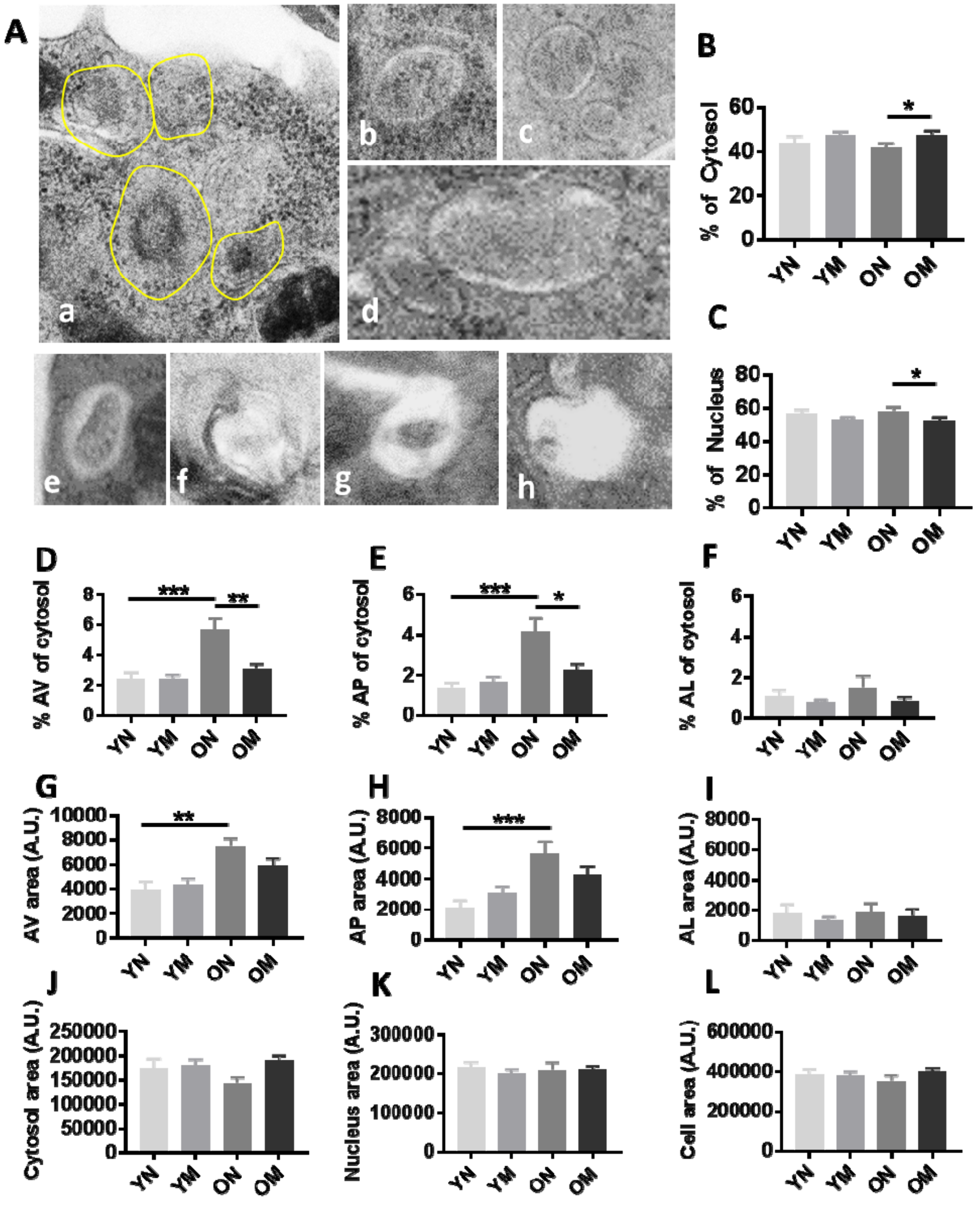

Supplementary Figure 4. (**A**) Examples of autophagic vacuolar compartments in $CD4^+$T cells from older individuals. (**a**) Accumulation of autophagosomes, (**b**–**e**) autophagosomes containing sequestered mitochondria, and (**f**–**h**) examples of autolysosomes at different stages of maturation. (**B**) In older (O) donors, cytosol percentage was higher in memory (M) $CD4^+$T cells than naive (N) $CD4^+$T cells (**p*=0.0458). There was no difference in younger (Y) donors. (**C**) Accordingly in older donors, the nucleus percentage was higher in naive $CD4^+$T cells compered to memory cells (**p*=0.0458). (**D**) The autophagic vacuoles (AV) and also (**E**) autophagosomes (AP) percentage in the cytosol were higher in naïve $CD4^+$T cells from older compared to younger donors (****p*=0.0004, ****p*=0.0002, respectively). Also the AV and AP percentage in the cytosol were higher in naïve $CD4^+$T cells compared to memory $CD4^+$T cells in older donors (***p*=0.0027, **p*=0.0116, respectively). There was no difference in younger donors. (**F**) There were no significant differences in AL percentage in the cytosol in either cell type or between young and older donors. (**G**) AV area and (**H**) AP area were higher in naïve $CD4^+$T cells from older donors than naive $CD4^+$T cells from younger donors (***p*=0.0014, ****p*=0.0005, respectively). (**I**–**L**) AL, cytosol, nucleus, and cell area were similar in naïve and memory $CD4^+$T cells from older and younger participants. All *p*-values were calculated by Student's t-test using GraphPad PRISM 7 software. Error bars reflect the standard error of the mean (±SEM). (**G**–**L**) Arbitrary Unit (A.U.). N = 5 young, 4 old donors.

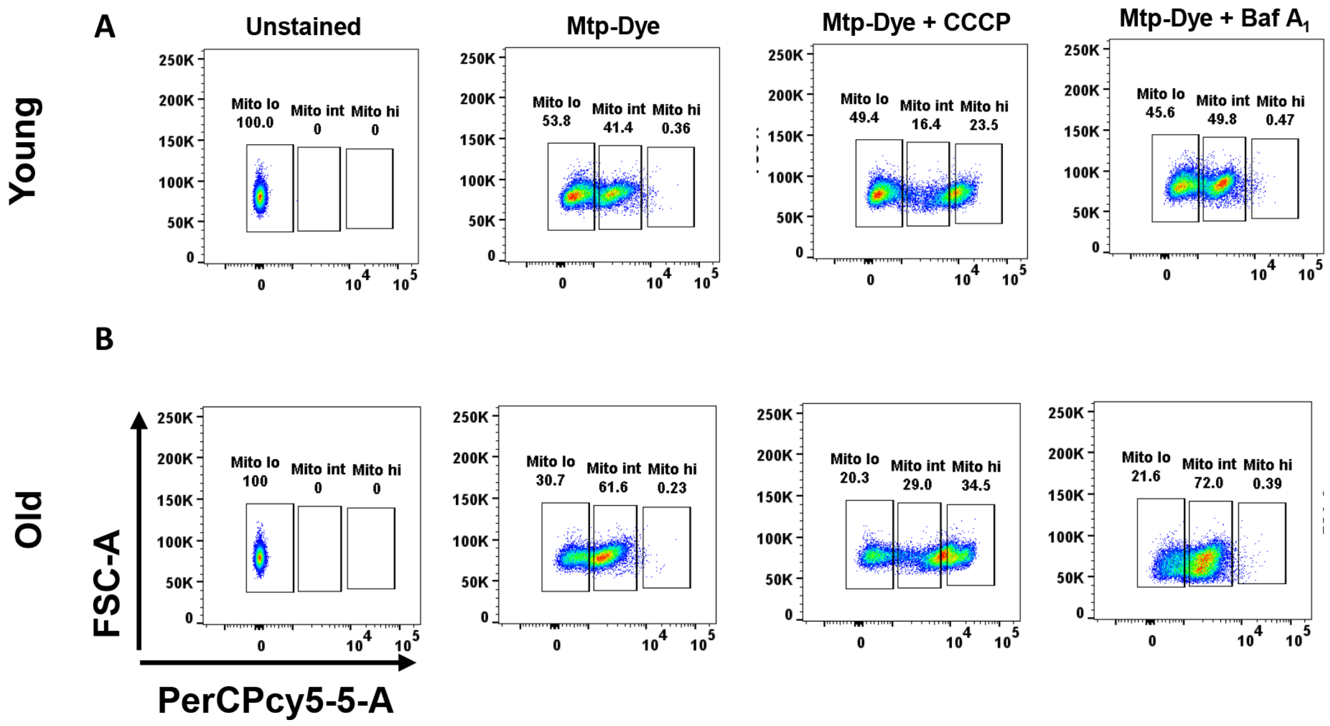

Supplementary Figure 5. Dot plot examples from (**A**) one young and (**B**) one old donor showing the percentage of gated cells for four treatment conditions (Column 1: Unstained, Column 2: Mtphagy (Mtp) Dye, Column 3: Mtp-Dye with CCCP treatment, Column 4: Mtp-Dye with bafilomycin A_1(Baf A_1) treatment. Percentages are shown for gates corresponding to low ('lo'), intermediate ('int'), and high ('hi') fluorescence. N = 12 young, 12 old donors.
