# Supplementary material for: Age-associated changes in human CD4+ T cells point to mitochondrial dysfunction consequent to impaired autophagy
Source: Aging (Albany NY). 2019 Nov 9;11(21):9234–63. doi: 10.18632/aging.102438 (PMC6874450; doi:10.18632/aging.102438)
Supplement: Supplementary Tables [file aging-11-102438-s001.pdf]

SUPPLEMENTARY TABLES

Supplementary Table 1. Sample donors for four different iTRAQ-8plex experiments used to determine protein expression in $CD4^+$ T cells.

iTRAQ Subjects								
iTRAQ	iTRAQ 1		iTRAQ 2		iTRAQ 3		iTRAQ 4	
Reagents	Donor ID	Age	Donor ID	Age	Donor ID	Age	Donor ID	Age
113	ND70	30	†ND73	21	ND74	34	*ND75	21
114	†ND73	21	ND74	34	†ND73	21	BL57	70
115	BL36	74	ND74	34	ND72	34	†ND73	21
116	BL59	75	BL46	74	BL83	70	ND74	34
117	BL49	77	BL23	73	BL76	71	BL31	71
118	*BL43	74	BL48	73	BL56	72	BL42	72
119	BL46	76	BL59	77	BL67	73	ND71	70
121	BL54	82	BL85	68	BL74	83	BL45	76

Donor ID letters indicate sample from either BLSA participant (BL) or normal donor (ND) followed by anonymized donor number.
$CD4^+$ T cell cytoplasmic extracts used in the analysis were from four young and 18 old male donors (*two samples [BL43 and ND75] were not analyzed due to technical concerns).
†One common reference sample (ND73, yellow box) was used in all four different iTRAQ 8plex experiments to help in normalization among iTRAQ runs.
Some samples were used more than once as technical controls both within an iTRAQ 8plex experiment (as with sample ND74) and between iTRAQ 8plex experiment (as with samples BL59, BL46, and ND74).
Different ages with the same sample donor (BL59 and BL46) indicate blood collection from two different visits (technical control).

Supplementary Table 2. Cellular origin of proteins with higher expression in CD4$^+$ T cells of older donors compared to younger donors as assessed by iTRAQ and mass spectrometry (LC-MS-MS).

Oxidative phosphorylation proteins	Electron transport chain
DHSA	Complex 2
DHSB	Complex 2
COX41	Complex 4
CX7A2	Complex 4
COX5A	Complex 4
COX7B	Complex 4
COX7C	Complex 4
COX2	Complex 4
NDUA4	Complex 4
ATPA	Complex 5
ATPB	Complex 5
ATP6	Complex 5
ATP5H	Complex 5
ATP5J	Complex 5
ATPO	Complex 5
ATPD	Complex 5

Membrane proteins	Subcellular location
GPDM	mitochondrial
NB5R3	cytoplasmic, mitochondrial and other membranes
MPCP	mitochondrial
CYB5B	mitochondrial
BCL2	mitochondrial, nuclear, endoplasmic reticulum
STML2	mitochondrial
ADT1	mitochondrial inner membrane, multipass membranes

Non-Mitochondrial proteins	Subcellular location
HNRPL	nuclear
SUN2	nucleus inner membrane, endosome membrane
LMNB1	nuclear
PGRC2	membrane binding protein
ITAM	membrane
DPM1	membrane

Supplementary Table 3. Sample donors for performing microarray gene-expression analysis using RNA isolated from CD4$^+$ T cells obtained from 33 donors (24 men and 9 women of which 8 were young [25–38 years old] and 25 old [70–82 years old]).

Microarray Subjects				
**Donor ID**	**Age**	**Sex**	**Y/O**	
ND29	25	M	Y	8 Young
ND703	26	F	Y	5M/3F
ND60A	29	F	Y	
BL10	30	M	Y	
ND340	30	M	Y	
ND691	32	M	Y	
ND554	33	M	Y	
BL71	38	F	Y	
BL47	70	F	O	25 Old
BL17	70	M	O	19M/6F
BL48	70	M	O	
BL57	70	M	O	
BL83	70	M	O	
ND31	71	F	O	
BL23	71	M	O	
BL31	71	M	O	
BL43	71	M	O	
BL22	72	M	O	
BL42	72	M	O	
BL56	72	M	O	
BL50	73	F	O	
BL7	73	M	O	
BL26	73	M	O	
BL36	74	M	O	
BL46	74	M	O	
BL13	75	F	O	
BL9	75	M	O	
BL63	75	M	O	
BL39	79	M	O	
BL40	80	F	O	
BL2	81	F	O	
BL54	82	M	O	
BL82	82	M	O	

Donor ID letters indicate sample from either BLSA participant (BL) or normal donor (ND) followed by anonymized donor number. 8 Young (5M/3F), 25 Old (19M/6F).

Supplementary Table 4A. Sample donors for OROBOROS mitochondrial respiration analysis using CD4$^+$ T cells obtained from 14 men (22–93 y/o; 7 young, 7 old).

OROBOROS Subjects				
**Donor ID**	**Age**	**Sex**	**Y/O**	
TP22	22	M	Y	7 Young
TP23	22	M	Y	All M
TP21	23	M	Y	22 to 35
TP26	28	M	Y	
TP25	30	M	Y	
TP20	34	M	Y	
TP24	35	M	Y	
BL96	80	M	O	7 Old
BL97	80	M	O	All M
BL92	82	M	O	80 to 93
BL91	84	M	O	
BL93	85	M	O	
BL95	85	M	O	
BL94	93	M	O	

Supplementary Table 4B. Percentage of Naïve (N) and Memory [Central Memory (CM) and Effector Memory (EM)] CD4$^+$ cells.

Donor ID	Y/O	% of Naïve	% of Memory
TP22	Y	45	36
TP23	Y	47	33
TP21	Y	64	21
TP26	Y	59	24
TP25	Y	52	31
TP20	Y	38	37
TP24	Y	47	37
BL96	O	18	59*
BL97	O	55	26
BL92	O	26	48
BL91	O	40	38
BL93	O	53	27
BL95	O	48	29
BL94	O	38	41

*outlier.

Donor ID letters indicate sample from either BLSA participant (BL) or Tissue Procurement donor (TP) followed by anonymized donor number. Male (M), Female (F), Young (Y), Old (O).

Supplementary Table 5. Sample donors for analysis of $CD4^+$ naïve cells and $CD4^+$ memory cells from younger and older volunteers by transmission electron microscopy (TEM) looking at mitochondrial numbers, mitochondrial areas, and also autophagosomes, autolysosomes, and autophagic vacuoles.

TEM Subjects				
**Donor ID**	**Age**	**Sex**	**Y/O**	**Naïve/Memory**
ND83	22	F	Y	Naïve and Memory
ND84	26	F	Y	Naïve and Memory
ND82	27	M	Y	Naïve and Memory
ND78	31	M	Y	Naïve and Memory
ND79	34	F	Y	Naïve and Memory
ND76	70	M	O	Naïve and Memory
ND77	72	M	O	Naïve and Memory
BL98	76	M	O	Naïve and Memory
BL99	80	M	O	Naïve and Memory

5 Young (23 to 34): 5 Young Naïve and 5 Young Memory
5 Old (70 to 80): 5 Old Naïve and 5 Old Memory
Donor ID letters indicate sample from either BLSA participant (BL) or normal donor (ND) followed by anonymized donor number.
Male (M), Female (F), Young (Y), Old (O).

Supplementary Table 6. Sample donors for mitophagy detection analysis using $CD4^+$ T cells obtained from 24 men and women (23–90 y/o; 12 young, 12 old).

Donor ID	Age	Sex	Y/O
TP32	23	M	Y
TP34	23	M	Y
TP35	26	F	Y
TP28	28	F	Y
TP27	29	F	Y
BL108	31	F	Y
BL104	31	M	Y
TP31	32	M	Y
TP33	32	M	Y
TP30	34	F	Y
TP29	35	M	Y
BL110	38	F	Y
BL112	75	F	O
BL102	76	M	O
BL109	76	F	O
BL106	77	M	O
BL111	79	F	O
BL101	79	M	O
BL103	81	M	O
BL113	81	F	O
BL114	82	F	O
BL105	83	M	O
BL100	88	M	O
BL107	90	F	O

Donor ID letters indicate sample from either BLSA participant (BL) or Tissue Procurement donor (TP) followed by anonymized donor number.
Male (M), Female (F), Young (Y), Old (O).
